# A Manual of Modern Gastric Methods, Chemical, Physical, and Therapeutical

**Published:** 1900-09

**Authors:** 


**A Manual of Modern Gastric Methods, Chemical, Physical, and Therapeutical.** By A. Lockhart Gillespie, M.D. **With a Chapter upon the Mechanical Methods used in Young Children.** By John Thomson, M.D. Pp. x., 175. Edinburgh: Oliver and Boyd. 1899.

Considerable advance has been made in recent years in the study and treatment of gastric disorders, partly due to improved apparatus, but chiefly to the more accurate knowledge of the chemical changes taking place in the stomach during digestion gained by systematic examination of its contents, and also to the recognition of the importance of the movements and degree of distension of the organ during and in the intervals of digestion. The literature of the subject has become very extensive, and the practitioner has probably, whilst anxious to move with the times, not been able to keep up with it. Further, authors are apt to recommend their own especial methods, and it is difficult for the practitioner with but a limited opportunity for testing them to know which is the best for his purpose. A book such as this one, which gives an account in moderate compass of the chief advances made in the treatment of gastric disorders by one who is an acknowledged authority on the subject, and which at the same time gives clear directions as to the manner in which the methods recommended should be carried out, is just the very one required, and should meet with widespread success. It seems to us that Dr. Gillespie's work is admirably adapted to aid the practitioner in his work; it is not too long, it is clearly written, and it gives a sufficient account of those methods which will be found most useful in diagnosis, and of the indications which the information thus gained affords for treatment. Dr. John Thomson gives a useful chapter upon the mechanical measures advisable for use on children.

